# Brain iron accumulation affects myelin-related molecular systems implicated in a rare neurogenetic disease family with neuropsychiatric features

**DOI:** 10.1038/mp.2015.192

**Published:** 2016-01-05

**Authors:** M Heidari, D M Johnstone, B Bassett, R M Graham, A C G Chua, M J House, J F Collingwood, C Bettencourt, H Houlden, M Ryten, J K Olynyk, D Trinder, E A Milward

**Affiliations:** 1 School of Biomedical Sciences and Pharmacy, The University of Newcastle, Callaghan, NSW, Australia; 2 Bosch Institute and Discipline of Physiology, University of Sydney, Sydney, NSW, Australia; 3 School of Biomedical Sciences and Curtin Health Innovation Research Institute - Biosciences, Curtin University of Technology, Bentley, WA, Australia; 4 School of Medicine and Pharmacology, University of Western Australia, Fiona Stanley Hospital, Murdoch, WA, Australia; 5 Harry Perkins Institute of Medical Research, Murdoch, WA, Australia; 6 School of Physics, University of Western Australia, Crawley, WA, Australia; 7 Warwick Engineering in Biomedicine, School of Engineering, University of Warwick, Coventry, UK; 8 Department of Molecular Neuroscience, UCL Institute of Neurology, London, UK; 9 Department of Clinical and Experimental Epilepsy, UCL Institute of Neurology, London, UK; 10 Department of Medical and Molecular Genetics, King's College London, London, UK; 11 Institute for Immunology and Infectious Diseases, Murdoch University, Perth, WA, Australia; 12 Department of Gastroenterology and Hepatology, Fiona Stanley Hospital, The University of Western Australia, Murdoch, WA, Australia; 13 Department of Gastroenterology and Hepatology, Fremantle Hospital, Fremantle, WA, Australia

## Abstract

The ‘neurodegeneration with brain iron accumulation' (NBIA) disease family entails movement or cognitive impairment, often with psychiatric features. To understand how iron loading affects the brain, we studied mice with disruption of two iron regulatory genes, *hemochromatosis* (*Hfe*) and *transferrin receptor 2* (*Tfr2*). Inductively coupled plasma atomic emission spectroscopy demonstrated increased iron in the *Hfe*^−/−^ × *Tfr2*^*mut*^ brain (*P*=0.002, *n* ≥5/group), primarily localized by Perls' staining to myelinated structures. Western immunoblotting showed increases of the iron storage protein ferritin light polypeptide and microarray and real-time reverse transcription-PCR revealed decreased transcript levels (*P*<0.04, *n* ≥5/group) for five other NBIA genes, *phospholipase A2 group VI*, *fatty acid 2-hydroxylase*, *ceruloplasmin*, *chromosome 19 open reading frame 12* and *ATPase type 13A2*. Apart from the ferroxidase ceruloplasmin, all are involved in myelin homeostasis; 16 other myelin-related genes also showed reduced expression (*P*<0.05), although gross myelin structure and integrity appear unaffected (*P*>0.05). Overlap (*P*<0.0001) of differentially expressed genes in *Hfe*^−/−^ × *Tfr2*^*mut*^ brain with human gene co-expression networks suggests iron loading influences expression of NBIA-related and myelin-related genes co-expressed in normal human basal ganglia. There was overlap (*P*<0.0001) of genes differentially expressed in *Hfe*^−/−^ × *Tfr2*^*mut*^ brain and post-mortem NBIA basal ganglia. *Hfe*^−/−^ × *Tfr2*^*mut*^ mice were hyperactive (*P*<0.0112) without apparent cognitive impairment by IntelliCage testing (*P*>0.05). These results implicate myelin-related systems involved in NBIA neuropathogenesis in early responses to iron loading. This may contribute to behavioral symptoms in NBIA and hemochromatosis and is relevant to patients with abnormal iron status and psychiatric disorders involving myelin abnormalities or resistant to conventional treatments.

## Introduction

Abnormalities in brain iron may contribute to various psychiatric disorders, including major depression, bipolar disorder and autism,^1, 2, 3^ and to diseases such as Alzheimer's disease, Parkinson's disease and Huntington's disease, that can have psychiatric features.^4, 5^ Psychiatric symptoms are also a feature of the rare neurogenetic disease family termed ‘neurodegeneration with brain iron accumulation' (NBIA), characterized by iron accumulation in the basal ganglia.^6^ Patients usually have movement disorders such as dystonia, spasticity or parkinsonism, often accompanied by neuropsychological and psychiatric features, including delusions, hallucinations, personality changes with emotional lability, depression or violent outbursts, impulsivity, hyperactivity, poor attention span or cognitive impairment.^6, 7, 8, 9^

Although NBIAs are typically severely debilitating and sometimes fatal,^4, 5^ the more common iron loading disease hemochromatosis is generally less severe and specific neuropsychiatric symptoms are not widely recognized features.^4^ However, several case reports document specific psychiatric conditions such as bipolar, major depressive and psychotic disorders in small numbers of hemochromatosis patients, including several instances where patients resistant to conventional psychiatric treatments experienced partial or complete recovery following phlebotomy or chelation treatment.^10^ Larger studies assessing iron are almost nonexistent, but one study of psychiatric clinic outpatients estimated a systemic iron overload prevalence of 1%, associated with an unexpectedly high rate of diagnoses of bipolar affective disorder (80%) and, without exception, atypical resistance to psychiatric treatment.^2^

The mechanisms by which iron perturbation affects the brain are poorly understood, mainly because of lack of good models. Past rodent models typically examined short-term iron loading using injection or iron-supplemented diet,^4^ whereas human iron loading disorders usually involve slow iron accumulation over extended periods, with damage often only apparent later in life.^4^

Genetically modified mice provide better models of chronic, progressive human iron loading. We and others have investigated the brain iron phenotype of mice with disruption of the *Hfe* gene (*Hfe*^−*/*−^) or the transferrin receptor 2 gene (*Tfr2*^*mut*^), the iron regulatory genes causatively associated with hemochromatosis.^11, 12, 13^ However, although these models display chronic systemic iron loading, brain iron content remained unchanged at all ages investigated,^11, 12^ as also reported for a related *Hfe* H67D mutation ‘knock-in' mouse model.^14^

A new genetic mutant mouse model with simultaneous disruption of the *Hfe* and the *Tfr2* genes (*Hfe*^−/−^ × *Tfr2*^*mut*^) on an AKR background shows signs of early liver damage in the form of fibrosis, equivalent to mild clinical hemochromatosis.^15^ Hemochromatosis in patients with dual mutations in these two genes is typically of earlier onset but otherwise often clinically indistinguishable from hemochromatosis caused by either mutation alone.^16, 17, 18, 19^

In this study, *Hfe*^−/−^ × *Tfr2*^*mut*^ mice fed a short-term high-iron diet, to maximize iron phenotype, showed increased brain iron loading, the first such demonstration in a hemochromatosis model, accompanied by hyperactivity and other behavioral changes. (As previously reported, AKR wild-type control mice on this diet do not show brain iron increases.^20^) Iron accumulated preferentially in myelinated tracts throughout the brain and in a subset of myelin-associated oligodendroglial cells, with few if any neurons, astrocytes or microglia showing discernible iron staining. This was accompanied by numerous molecular changes, including alteration of six NBIA-associated transcript or protein species. As far as we are aware, this is the first demonstration that brain iron loading influences the expression of a suite of genes causatively linked to NBIA.

We found that most of the NBIA-linked genes with altered expression in response to brain iron loading are directly or indirectly related to myelin and that several other myelin-related genes also show expression changes in the mouse model. There was significant correspondence between genes that are differentially expressed in mouse brain in response to increased iron loading and a gene co-expression set from human brain basal ganglia that is enriched for both NBIA-related genes and genes related to myelin or oligodendrocytes. There was also overlap with genes identified as differentially expressed in basal ganglia from NBIA cases that were significantly enriched for various myelin-related ontologies. As myelin changes are hypothesized to contribute to many psychiatric disorders, including schizophrenia, bipolar disorder and depression,^21, 22^ these findings raise the possibility of a relationship between iron, myelin and the psychiatric symptoms observed in some NBIA cases.

## Materials and methods

### Mice and tissue collection

Cross-breeding of homozygous *Hfe*^−/−^ mice and *Tfr2*^*mut*^ mice on an AKR genetic background^23, 24^ generated mice homozygous for both mutations.^15^ The AKR background manifested stronger iron loading than other genetic backgrounds to which it was compared.^25, 26^ Unless otherwise stated, all experiments were performed on male mice. Mice were maintained *ad libitum* on a standard diet containing ∼0.02% iron. To maximize iron status, *Hfe*^−/−^x*Tfr2*^*mut*^ mice were switched to an iron-supplemented diet containing 2% carbonyl iron (Sigma Aldrich, St Louis, MO, USA) for 3 weeks before killing. At 13 weeks of age, mice were killed under anesthesia (50 mg kg^−1^ ketamine, 10 mg kg^−1^ xylazine; Troy Laboratories, Pty Ltd, Smithfield, NSW, Australia). Mice were perfused transcardially with isotonic saline, brains excised, snap-frozen in liquid nitrogen and stored at −80 °C. All protocols were approved by the Animal Ethics Committees of the Universities of Western Australia and Sydney.

### Measurement of iron and ferritin

Homogenized brain tissue (150 mg) from wild-type and *Hfe*^−/−^ × *Tfr2*^*mut*^ mice (*n ≥*5/group) was digested with concentrated nitric acid (69%, 10 ml, 60 °C). After solubilization, samples were heated to 95 °C until volume was reduced to ~1 ml. Samples were diluted to ~8 ml in 1% nitric acid and iron, cadmium, copper, manganese, lead, selenium and zinc measured by inductively coupled plasma atomic emission spectroscopy at the Marine and Freshwater Research Laboratory, Murdoch University, Perth, Australia.

Ferritin and β-actin protein levels were assessed by western immunoblotting as previously described^12^ (see Supplementary Table 1 for antibody details).

### RNA microarray preparation and analysis

Total RNA was isolated from *Hfe*^−/−^ × *Tfr2*^*mut*^ and wild-type mice (*n* ≥4/group) using TRI reagent (Ambion) and then purified and concentrated using the RNeasy MinElute Kit (QIAGEN, Hilden, Germany) and quantified by spectrophotometry. RNA samples were biotin labeled and amplified using the TotalPrep RNA Amplification Kit (Ambion, Austin, TX, USA). cRNA was hybridized onto Sentrix MouseRef-8 v2 Expression BeadChips (Illumina, San Diego, CA, USA) and scanned using the Illumina BeadArray Reader and BeadScan software (V2.2.7). After data preprocessing with the GenomeStudio Gene Expression Module (Illumina, v2010.3) and background subtraction and normalization (Average or Cubic Spline), data were filtered to remove genes not expressed above background levels using a detection *P-*value filter of 0.01, as recommended by Illumina. Cluster analysis confirmed similarity of global gene expression profiles between the individual samples in each group and hence all samples were included in the final analyses. Differential expression analysis was then performed using GenomeStudio or GeneSpring GX 7.3 (Agilent Technologies, Santa Clara, CA, USA). No multitest correction was applied for discovery-driven experiments.^27^ All analyses were performed on both the union and the intersection gene lists generated from the four data sets (that is, Average- or Cubic Spline-normalized probes analyzed by GenomeStudio and Average- or Cubic Spline-normalized probes analyzed by GeneSpring).^28^ Unless otherwise stated, all reported outcomes were observed for both gene lists. To validate microarray data, real-time reverse transcription-PCR was performed as previously described.^11^ For primer sequences see Supplementary Table 2. Transcript levels of genes of interest were normalized to the geometric mean expression of *Actb*, *Gapdh*, *Hprt* and *Rpl13a*. Enriched pathways and ontologies were identified by DAVID (http://david.abcc.ncifcrf.gov/home.jsp), KEGG (http://www.genome.jp/kegg/) and GATHER (http://gather.genome.duke.edu/).

### Comparison with human normal and NBIA basal ganglia transcriptomes

Differentially expressed genes in the *Hfe*^−/−^ × *Tfr2*^*mut*^ brain were compared with a set of genes showing expression correlations in human basal ganglia and enriched for both NBIA-related genes and genes related to myelin and oligodendrocytes. This gene set was identified by unsupervised weighted gene co-expression network analysis of whole-transcriptome profiles from 10 brain regions in neuropathologically normal adult brain (*n*=101).^29^

In a separate analysis, differential gene expression was assessed in post-mortem basal ganglia of two clinicopathologically confirmed NBIA cases of unknown genetic subtype (male 66 years and female 81 years) from the Canadian Brain Tissue Bank, University of Toronto, compared with two age- and gender-matched adults with no diagnosed neurological conditions from the Newcastle Brain Tissue Resource, University of Newcastle, UK. Tissue was obtained with fully informed consent and the study approved by the Human Research Ethics Committee of the University of Newcastle, Australia (H-2010-1219). Total RNA was isolated and prepared for microarray as previously described.^11, 12^ Microarrays were performed using HumanHT-12 v4 Expression BeadChips (Illumina). Following Cubic Spline normalization in GenomeStudio (Illumina, v2010.3), genes were considered differentially expressed if the fold change of the mean NBIA signal relative to mean control signal was at least 1.5. Pathways were analyzed by DAVID and the gene list also compared with the NBIA- and myelin-related gene set identified from the human basal ganglia co-expressed gene list described above,^29^ with significant overlap determined by χ^2^ testing (*P*<0.05). The array data from the *Hfe*^−/−^ × *Tfr2*^*mut*^ mice (GSE70431), NBIA basal ganglia (GSE70430) and normal brain (GSE46706) have been deposited for public access at Gene Expression Omnibus.

### Histology and immunohistochemistry

**Figure 1:**
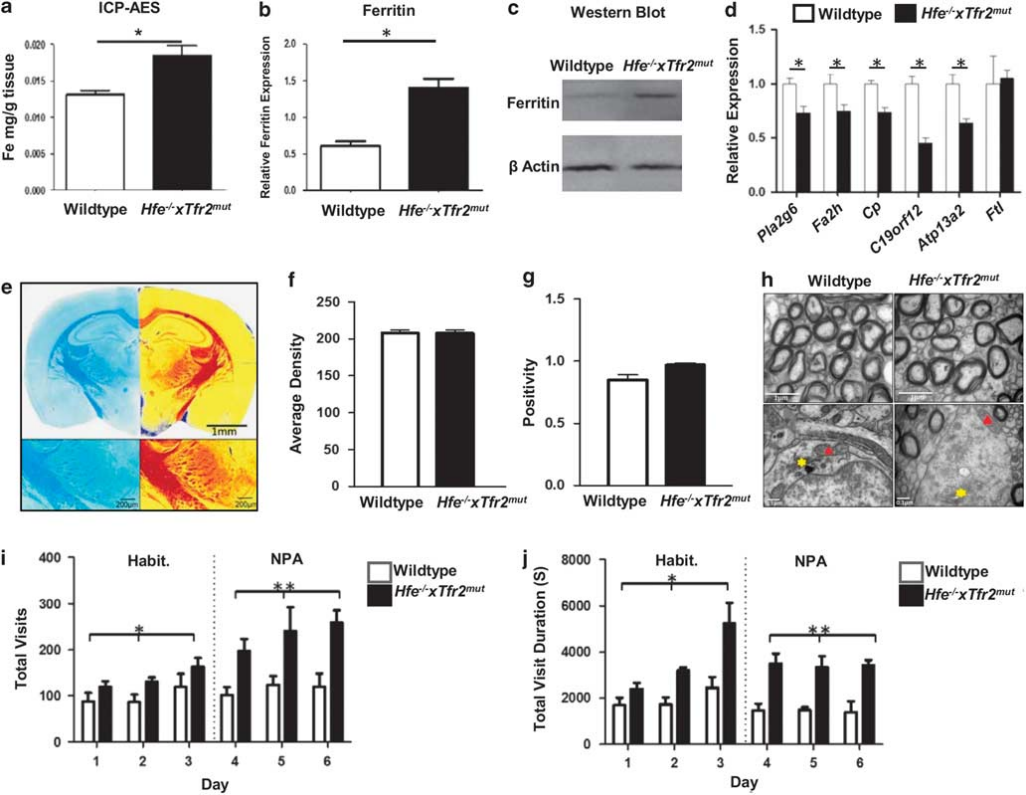
(**a**) Whole-brain iron levels by inductively coupled plasma atomic emission spectroscopy (ICP-AES). Mean±s.e.m., Student's *t*-test *P=0.002, *n ≥*5/group. (**b** and **c**) Immunoblot analysis of ferritin relative to β-actin. Mean±s.e.m., Student's *t*-test *P=0.0005, *n ≥*5 per group. (**d**) Real-time reverse transcription-PCR (RT-PCR) validation of changes in transcript levels for selected neurodegeneration with brain iron accumulation (NBIA) genes. Mean±s.e.m., Student's *t*-test *P<0.04, *n*=7/group. (**e**) Luxol Fast Blue staining (left panels) was analyzed by the Positive Pixel Count algorithm (right panels) that grades areas as high (red), moderate (orange) or weak (yellow) positive or negative (blue) staining. (**f** and **g**) Average density (total intensity of all positive pixels divided by total number of positively stained pixels) and positivity (number of positive pixels divided by the total pixels; measures proportion of brain area staining positive) of Luxol Fast Blue staining for myelin were unchanged. Mean±s.e.m., Mann–Whitney test *P*>0.05, *n≥*4/group. (**h**) Electron micrographs of myelinated axons (top) and glial cell organelles (bottom) in the corpus callosum; oligodendrocyte mitochondria (red arrows) and Golgi apparatus (yellow stars). (**i** and **j**) Activity measures (number of visits and duration of visits per test group per day) in the habituation (Habit.) and nose-poke adaptation (NPA) phases of IntelliCage testing. Mean±s.e.m., two-way ANOVA (**i**) *P<0.0112 (Habit.) and **P<0.0004 (NPA); (**j**) *P<0.0007 (Habit.) and **P<0.0001 (NPA), *n ≥*6/group.

Mouse brains (*n ≥*4/group) were perfusion fixed (4% paraformaldehyde) and cryosectioned (20 μm). Iron was detected by 3,3′-diaminobenzidine-enhanced Perls' stain.^30^ Antibodies for immunohistochemical analysis of myelin and cell identification are detailed in Supplementary Table 1. Myelin quantification (*n* ≥4 mice/group) was performed between anterioposterior Bregma coordinates of −1.46 to −2.18. Triplicate sections were stained with Luxol Fast Blue (IHC World, Woodstock, MD, USA) and digitized with the Aperio Digitial Pathology System (Leica Biosystems, Wetzlar, Hesse, Germany). Corpus callosum width and area were measured as described elsewhere^31, 32^ and Luxol Fast Blue staining density quantified with the Aperio ImageScope Positive Pixel Count tool (Version 12.1.0.5029), as illustrated in Figure 1e (for specific algorithm parameters see Supplementary Table 3).

### Transmission electron microscopy

To determine whether changes in myelin ultrastructure accumulated later in life, ultrathin sections (70–90 nm) of corpus callosum around the midline (−1.46 to −2.18 Bregma) were prepared from 2 male and 2 female mice per group at 9–11 months of age using published methods.^33^ Ten randomly selected fields per animal were imaged on a JEOL JEM-1200EXII (Tokyo, Japan) transmission electron microscope. Number of myelinated axons per 100 μm^2^, cross-sectional area, thickness of myelinated axons (*n >*100) and ‘g-ratio' of axon thickness relative to myelin sheath thickness were obtained using ImageJ (National Institutes of Health, Bethesda, MA, USA).

### Behavioral study

Cognitive function was tested with the IntelliCage system (NewBehavior AG, Zürich, Switzerland) using a published place learning protocol comprising 3 days of habituation and 3 days of nose-poke adaptation, followed by 5 days of place learning and 5 days of reversal of place learning.^34^ Groups of 9-month-old wild-type and *Hfe*^−*/*−^ *× Tfr2*^*mut*^ mice (*n*=6/group, equal numbers of male and female) were housed in separate IntelliCages. Two female wild-type mice were excluded during the experiment because of inadequate drinking activity, in accordance with IntelliCage criteria.^34^

## Results

### Brain iron status

As shown in Figure 1a, total iron levels, measured by inductively coupled plasma atomic emission spectroscopy, were higher in whole-brain homogenate from *Hfe*^−*/*−^ *× Tfr2*^*mut*^ mice than wild-type mice (fold change 1.42, *P*=0.002, *n* ≥5/group). There were no significant differences (*P*>0.05) for copper, zinc and manganese, whereas cadmium, lead and selenium were below detection limits.

Western immunoblotting of whole-brain homogenate and quantification by densitometry revealed 2.3-fold increased levels of ferritin protein (*P*=0.0005, *n ≥*5/group) in the *Hfe*^−*/*−^ *× Tfr2*^*mut*^ mouse brain relative to wild-type (Figures 1b and c and Table 1).

### Microarray analysis and NBIA-related gene expression

In total, 12 318 probes passed the detection *P*-value cutoff of 0.01 following normalization and were retained in the analysis. The union list generated by normalization and differential expression analysis contained 2615 probes, whereas the intersection set contained 761 probes. Most gene expression changes (95.7%) were below twofold in magnitude.

Functional classification using DAVID and GATHER identified 11 significantly overrepresented pathways (*P*<0.05), including pathways related to oxidative phosphorylation and neurodegenerative diseases, notably Parkinson's disease, Alzheimer's disease, Huntington's disease and amyotrophic lateral sclerosis (Supplementary Figure 1). Another overrepresented pathway was mitogen-activated protein kinase signaling. Intriguingly, the gene showing the greatest expression change in this pathway was *phospholipase A2, group VI* (*Pla2g6;* fold change −1.60, *P*=0.010). The NBIA *PLA2G6*-associated neurodegeneration is caused by mutations in this gene.^35^

Of the 10 genes with mutations causatively linked to NBIA, 5 showed reduced transcript levels in *Hfe*^−*/*−^ *× Tfr2*^*mut*^ brain relative to wild-type (Table 1: *Pla2g6*; *fatty acid 2-hydroxylase* (*Fa2h*); ceruloplasmin (*Cp*); chromosome 19 open reading frame 12 (*C19orf12*); ATPase type 13A2 (*Atp13a2*)). The array did not include effective probes for *ferritin light polypeptide* (*Ftl*), another NBIA-linked gene, but no significant difference in *Ftl* transcript levels was observed by real-time reverse transcription-PCR, providing evidence that the increased levels in ferritin protein noted above reflect post-transcriptional regulation, as typically occurs in many iron loading scenarios.^36^ The χ^2^ testing revealed that significantly more NBIA-linked genes showed altered expression than would be predicted by chance alone (*P*=0.009). Transcripts for four other NBIA-related genes (Table 1) did not differ significantly. All results were confirmed by real-time reverse transcription-PCR (Figure 1d).

### Expression changes of genes related to myelin

Except for *Cp*, which encodes a ferroxidase,^37^ none of the NBIA genes with altered expression have well-established roles in iron homeostasis. However, one common feature is that all are associated with NBIA phenotypes showing pathological features of demyelination.^8, 38, 39, 40, 41^ The proteins encoded by these genes are still not well characterized but there is mounting evidence for myelin-related functions^41, 42, 43^ (Supplementary Table 4). Another 16 transcripts relating to myelin also showed decreased levels in *Hfe*^−*/*−^ *× Tfr2*^*mut*^ brain (all *P*<0.05), including transcripts for the iron transporter transferrin that is expressed in oligodendrocytes and is essential for myelination.^44^ No myelin-related transcripts showed significant increases (Supplementary Table 5).

### Comparison of gene expression changes in the *Hfe*^−*/*−^*× Tfr2*^*mut*^ brain with a human basal ganglia gene expression network relevant to NBIA and myelin

We compared gene expression changes in *Hfe*^−*/*−^ *× Tfr2*^*mut*^ brain with a network of genes showing expression correlations in normal human basal ganglia that is enriched for both NBIA-related genes and genes related to myelin and oligodendrocytes. There was significant overlap between this human network and genes differentially expressed in the *Hfe*^−*/*−^ *× Tfr2*^*mut*^ mouse brain (*P*<0.0001).

### Comparisons with gene expression in the basal ganglia of NBIA cases

The rarity of NBIA makes obtaining post-mortem brain samples difficult but we performed restricted array analysis of post-mortem basal ganglia from two clinicopathologically confirmed NBIA patients and matched controls. The χ^2^ analysis showed significant overlap of putative differentially expressed genes from NBIA brains and *Hfe*^−*/*−^ *× Tfr2*^*mut*^ mice brains (*P*<0.0001).

There were no consistent changes in levels of prominent iron-related transcripts in these cases, with levels of most iron-related transcripts appearing unaltered, including ferritin and ceruloplasmin transcripts, although transcripts for the ferroxidase hephaestin were identified as being downregulated in tissue from one NBIA patient but not the other. However, numerous myelin-related transcripts were identified as differentially expressed in both NBIA brains (Supplementary Table 6).

Pathway and ontology analysis, using DAVID, of genes identified in NBIA brains showed significant enrichment of several myelin-related ontologies, notably ‘myelin sheath', ‘adherens junction', ‘focal adhesion' and ‘fatty acid metabolic processes' (all *P*<0.05). Other pathways that were enriched in the set of common genes identified as differentially expressed in both *Hfe*^−*/*−^ *× Tfr2*^*mut*^ mouse and NBIA brains again included pathways relating to neurodegenerative diseases (specifically Alzheimer's disease, Parkinson's disease and Huntington's disease), oxidative phosphorylation and the mitogen-activated protein kinase pathway (Supplementary Figure 1).

### Iron accumulates in myelin tracts and myelin-associated cells

**Figure 2:**
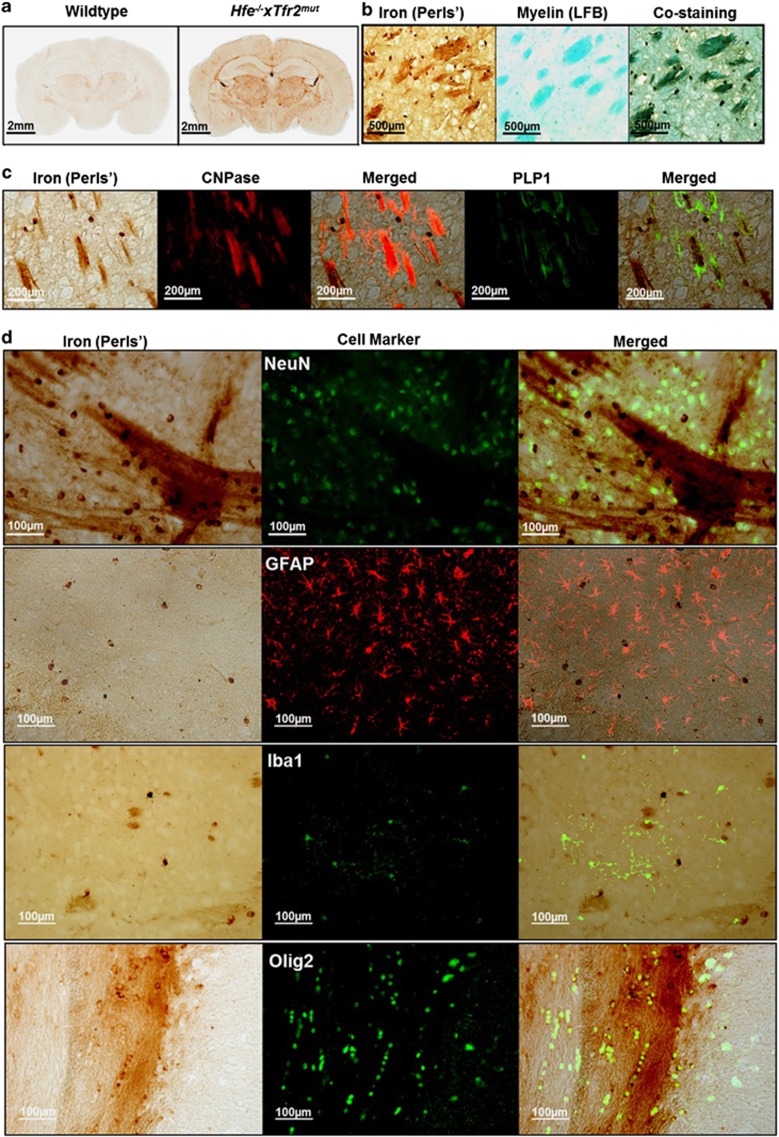
(**a**) Iron distribution in wild-type and *Hfe*^−*/*−^ *× Tfr2*^*mut*^ mouse brain at 3 months of age by 3,3′-diaminobenzidine (DAB)-enhanced Perls' staining. Iron is predominantly localized to myelinated areas throughout the brain, including the corpus callosum and deep white matter of the basal ganglia. (**b**) Colocalization of staining for iron and Luxol Fast Blue (LFB) staining for myelinated patches in adjacent coronal sections of *Hfe*^−*/*−^ *× Tfr2*^*mut*^ caudate putamen. (**c**) Colocalization of iron and myelin tracts immunolabeled with 2′,3′-cyclic nucleotide 3′-phosphodiesterase (CNPase) or proteolipid protein 1 (PLP1). (**d**) Colabeling of iron with immunolabeling for neurons (NeuN), astrocytes (GFAP), microglia (Iba1) and oligodendroglia (Olig2). Digitally merged images show iron staining colocalizes with some but not all Olig2-positive oligodendroglia but few if any of the other cell types examined.

Iron labeling with 3,3′-diaminobenzidine-enhanced Perls' stain and Luxol Fast Blue myelin staining showed that iron accumulates preferentially in myelinated tracts throughout the brain (Figures 2a–c) and in a subset of myelin-associated oligodendroglial lineage cells expressing the oligodendrocyte transcription factor Olig2 (Figure 2d). Few, if any, neurons, astrocytes or microglia showed high levels of iron staining (Figure 2d). Myelinated structures and iron-loaded oligodendroglial cells generally appeared morphologically normal (Figures 1e–h and 2). No differences were observed in measures of total myelin by Luxol Fast Blue staining density, corpus callosum width and area or measures of myelin ultrastructure from electron micrographs, including axon cross-sectional area, g-ratio and number of myelinated axons (all *P*>0.05).

### Cognitive and behavioral testing

As shown in Figures 1i and j, *Hfe*^−*/*−^ *× Tfr2*^*mut*^ mice showed hyperactivity in the habituation and nose-poke adaptation phases of IntelliCage testing, with increased numbers of exploratory visits to cage monitoring areas (two-way analysis of variance *P*<0.0112 and *P*<0.0004 respectively), in conjunction with significantly increased visit durations in these phases (two-way analysis of variance *P*<0.0007 and *P*<0.0001 respectively). There was no effect of genotype on cognitive function assessed by performance in the place learning task or reversal of place learning task (two-way analysis of variance *P*>0.05).

## Discussion

This study demonstrates that increased iron levels in the *Hfe*^−*/*−^ *× Tfr2*^*mut*^ mouse brain are accompanied by decreased expression of a suite of NBIA-linked genes in conjunction with changes in mouse behavioral activity patterns. Iron was localized primarily to myelinated structures and was also present in a subset of myelin-associated oligodendroglial cells. Aside from *Ftl*, no NBIA-related transcripts show altered expression in either the *Hfe*^−*/*−^ or *Tfr2*^*mut*^ single mutant models that do not have increased brain iron.^11, 12^ Of the six NBIA-linked genes (*Pla2g6*, *Fa2h*, *Cp*, *C19orf12*, *Atp13a2* and *Ftl)* with altered transcript or protein expression in the *Hfe*^−*/*−^ *× Tfr2*^*mut*^ brain, only *Cp* and *Ftl* are directly implicated in iron homeostasis, encoding a ferroxidase important in iron export^37^ and an iron storage protein, respectively.^45^ However, the remaining genes have putative functions relating to myelin, an important site of brain iron storage.^41, 42, 43, 46, 47, 48^ In addition, we observed expression changes in other myelin-related genes in the iron-loaded mouse brain and enrichment of myelin-related ontologies in human NBIA brain, further supporting connections between iron, myelin and NBIA.

Our study provides evidence for ceruloplasmin gene regulation in response to iron levels, consistent with an earlier study in rats.^49^ This may help protect against excessive brain iron accumulation as moderate reductions in *Cp* transcripts could potentially reduce export of iron from cerebrovascular endothelial cells into the brain.^50, 51^ Ceruloplasmin deficiency is not usually associated with myelin changes but *FTL*-linked NBIA (neuroferritinopathy) often involves extensive myelin loss.^47^ Ferritin expression is typically regulated post-transcriptionally through the iron-responsive element/iron-regulatory protein system without requiring concomitant changes in transcript levels.^36^ The increased *Hfe*^−*/*−^ *× Tfr2*^*mut*^ brain ferritin protein levels we observed are consistent with an iron-responsive element/iron-regulatory protein system feedback mechanism triggered by increased brain iron levels that may help protect against damage by increasing intracellular iron storage capacity. The other NBIA genes showing expression changes do not have recognized iron-regulatory roles but may indirectly influence iron homeostasis, as all have connections to myelin, the main brain iron reservoir.^52^ All are associated with phenotypes that can involve myelin pathology^8, 38, 39, 40, 41^ and several encode proteins with functions relating to myelin.^41, 42, 43, 46^ Delayed myelination or disruption of myelin integrity occur in patients with *PLA2G6*-linked and FA2H-linked NBIA.^38, 39, 40, 53, 54^ Of the known NBIA genes, these have the strongest links to myelin and also showed the greatest changes in iron-loaded mouse brains. Products of pla2g6 activity are required for myelin homeostasis^55, 56, 57^ and FA2H is crucial for biosynthesis of myelin lipid components.^58, 59^ Patients with *C19orf12* and *ATP13A2* mutations show myelin reduction^8, 41, 60^ and we observed decreased expression of these genes in iron-loaded mouse brains. C19orf12 may be involved in fatty acid biogenesis^41^ and atp13a2 is a putative phospholipid flippase that translocates phospholipids across membranes^7, 61^ and may protect against iron cytotoxicity through effects on membrane permeability.^62^

The four NBIA-related genes that did not show expression changes are less strongly linked to myelin, although rare cases of *WDR45*-linked NBIA^63, 64^ and *DCAF17*-linked NBIA show subtle white matter changes.^65, 66^ The PANK2 protein, involved in coenzyme A metabolism,^67^ does not appear to be associated with clear myelin pathology.^68, 69^ We found no reports of myelin pathology in NBIAs involving *COASY* but there is little published neuropathology for these patients. The extent to which iron abnormalities cause myelin perturbations may depend partly on location. For example, in patients with *PANK2* mutations, iron accumulation appears mainly restricted to neurons and astrocytes, with sparing of oligodendrocytes,^68^ perhaps explaining why myelin pathology is not typically observed in these patients.

Myelin-related effects in the *Hfe*^−*/*−^ *× Tfr2*^*mut*^ brain were not limited to NBIA-associated genes but also included decreased levels of 16 other transcripts with roles in myelin formation, integrity and regulation (Supplementary Table 5). These included transcripts for transferrin that is important in both brain iron transport and myelination.^44, 70^ Although decreases in expression of myelin structural genes such as *Mobp*, which stabilizes myelin, may affect myelin integrity, some of the other downregulated myelin-related genes are negative regulators of oligodendrocyte survival, maturation or myelin production (see Supplementary Table 5 for *Rtn4*, *Tnfrsf21* and *Lingo1*), suggesting myelin repair or fresh synthesis may compensate for myelin abnormalities and help preserve myelin integrity.

Substantial myelin changes are not expected in our mouse model, as it represents early hemochromatosis that does not have brain abnormalities comparable to NBIA, where severe problems often occur in childhood. Even so, the extensive transcriptomic alterations provide evidence that the normal equilibrium of myelin-related systems is still perturbed in these mice, and this may increase vulnerability to stressors despite the absence of gross myelin changes. We do not yet know whether iron accumulates in myelin itself or, perhaps more likely, in the periaxonal space (or both) in this model, but in all these scenarios axonal health as well as myelin health could eventually be affected. This merits detailed future studies.

Further evidence that brain iron perturbation can affect myelin comes from a mouse model deficient in iron-responsive element-binding protein 2 (also called iron-regulatory protein 2).^71^ This model shows movement impairment and myelin-related changes accompanying abnormal brain iron deposition.^71^ However, NBIA transcript expression was not investigated in this study and, as far as we know, IRP2 mutations have not been associated with human disease.

The similarities between the mouse data and human brain gene co-expression networks suggest increased iron loading selectively influences expression of a suite of NBIA-related and myelin-related genes that are co-expressed in normal human basal ganglia. The overlap between the mouse and NBIA basal ganglia data also suggests analogies between molecular mechanisms affected by brain iron loading in mice and pathogenic mechanisms in NBIA patients and provides evidence for involvement of systems relating to myelin and oligodendrocytes. Excessive iron loading in or near myelinated tracts might act on oligodendroglia or myelin to cause pathogenic or compensatory changes in transcription of various genes, including some NBIA-related genes.

The mutant mice showed hyperactivity and other behavioral changes and are considered unusually aggressive by animal technicians, often requiring housing in separate cages to prevent fighting (DM Johnstone, unpublished observation). More studies are planned, including tests of basal ganglia function, as iron preferentially loads in basal ganglia myelinated tracts, and tests of aggression and mouse behavioral analogs of depression. In addition, as far as we are aware, psychiatric abnormalities have yet to be reported in the NBIA gene mouse models themselves, and hence additional stressors may be required to elicit abnormal psychiatric responses in relevant mouse models.

As discussed above, changes in our model are mild relative to NBIA. The mice do not show functional changes typically associated with neurodegenerative disease, with no clear cognitive or movement impairment at ages up to 9 months. However, we postulate that in NBIA patients with mutations in genes important in myelin or pathological peri-myelin iron accumulation, myelin damage may result. This may lead to release of iron from myelin stores, further raising iron levels in a positive feedback amplification cascade.

Numerous studies report perturbations of myelin and myelin-related systems in psychiatric disorders, with altered myelination during childhood and adolescence implicated in schizophrenia, autism and other conditions.^21, 22^ Myelin alteration can affect synapse formation and plasticity, conduction velocity and synchronic impulse trafficking between brain regions.^72^ This can affect normal mental performance^73^ and may contribute to psychiatric symptoms in disorders involving myelin abnormalities, including some NBIA subtypes.

No studies appear to have examined relationships between psychiatric symptoms and interdependent abnormalities in iron and myelin, or the molecular systems involved, although a few studies hint at possible links. For example, the shift from presymptomatic to clinical Huntington's disease, including the development of psychiatric symptoms, is distinguished by increased white matter iron with demyelination and axonal degeneration.^74^

In summary, our findings may provide insights into mechanisms contributing to behavioral symptoms in patients with NBIA or conditions such as hemochromatosis and transfusion-induced iron overload. The findings also have potential relevance to patients with myelin disorders such as multiple sclerosis as well as to patients with psychiatric disorders, particularly with suspected myelin abnormalities or resistant to conventional treatments. The small existing literature suggests neuropsychiatric patients with iron overload may respond well to venesection.^10^ Studies of NBIA patients with known mutations and of patients with neurobehavioral symptoms and evidence of brain or systemic iron loading will further illuminate relationships between iron and myelin.

## Figures and Tables

**Table 1:** NBIA-related genes investigated in the *Hfe* ^−*/*−^ *× Tfr2* ^*mut*^ mouse brain

*Gene name and symbol*	*Proposed roles of encoded protein*	*Fold change by array (*P-*value)*	*NBIA type*	*Psychiatric abnormality or cognitive impairment*	*Iron abnormality*	*Myelin abnormality*
*Phospholipase A2, group VI (Pla2g6)*	Fatty acid release from phospholipids	↓1.60 (0.010)	PLAN	Yes	MRI	MRI
*Fatty acid 2-hydroxylase (Fa2h)*	2-Hydroxy sphingolipid synthesis	↓1.41 (0.002)	FAHN	Yes	MRI	MRI, animal study
*Ceruloplasmin (Cp)*	Oxidation of Fe(II) to Fe(III)	↓1.35 (0.013)	Aceruloplasminemia	Yes	MRI, post-mortem, animal study	No
*Chromosome 19 open reading frame 12 (C19orf12)*	Fatty acid biogenesis	↓1.28 (0.023)	MPAN	Yes	MRI, post-mortem	MRI
*ATPase type 13A2 (Atp13a2)*	Ceramide synthesis in lysosome, phospholipid distribution in myelin	↓1.17 (0.047)	Kufor–Rakeb disease	Yes	MRI	Biopsy
*Ferritin, light polypeptide (Ftl)*	Iron storage	↑2.3 (0.0005)^a^	Ferritinopathy	Yes	MRI, animal model	Post-mortem
*Pantothenate kinase 2 (Pank2)*	Biosynthesis of CoA	NS	PKAN	Yes	MRI, post-mortem	No
*CoA synthase (Coasy)*	Biosynthesis of CoA from pantothenic acid	NS	CoPAN	Yes	MRI	NA
*WD repeat domain 45 (Wdr45)*	Autophagy	NS	BPAN	Yes	MRI	MRI
*DDB1 and CUL4 associated factor 17 (Dcaf17)*	Ubiquitin ligase	NS	Woodhouse–Sakati syndrome	Yes	MRI	MRI

Abbreviations: BPAN, β-propeller protein-associated neurodegeneration; FAHN, fatty acid hydroxylase-associated neurodegeneration; MPAN, mitochondrial membrane protein-associated neurodegeneration; MRI, magnetic resonance imaging; NA, no reported data available; NBIA, neurodegeneration with brain iron accumulation; NS, nonsignificant (*P*>0.05); PKAN, pantothenate kinase-associated neurodegeneration; PLAN, *PLA2G6*-associated neurodegeneration.

Data are presented as fold change and associated *P-*value is in brackets.

a By western immunoblotting.
