# Supplementary figures and images for: Brain iron accumulation affects myelin-related molecular systems implicated in a rare neurogenetic disease family with neuropsychiatric features
Source: Mol Psychiatry. 2016 Jan 5;21(11):1599–607. doi: 10.1038/mp.2015.192 (PMC5078858; doi:10.1038/mp.2015.192)

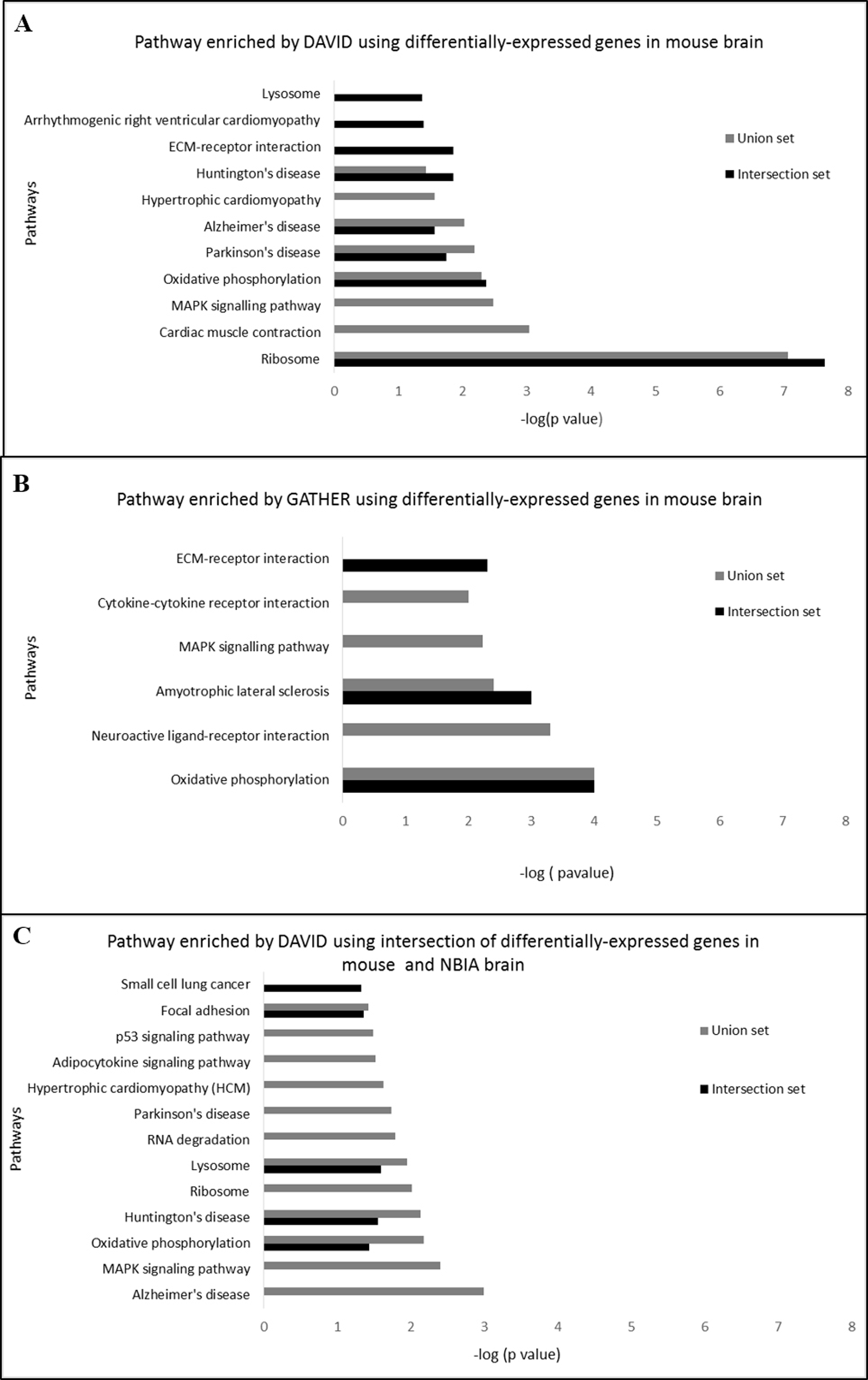

Supplement: Supplementary Figure S1 [file mp2015192x1.tif]
