# Supplementary material for: Brain iron accumulation affects myelin-related molecular systems implicated in a rare neurogenetic disease family with neuropsychiatric features
Source: Mol Psychiatry. 2016 Jan 5;21(11):1599–607. doi: 10.1038/mp.2015.192 (PMC5078858; doi:10.1038/mp.2015.192)
Supplement: Supplementary Information [file mp2015192x2.doc]

**ONLINE SUPPLEMENTS**

Supplementary Table 1. Antibody information

| **Antibody** | **Host species** | **Catalouge number** | **Company** | **Dilution factor** |
| --- | --- | --- | --- | --- |
| Ferritin | Rabbit | A0133 | DAKO | 1:2000 |
| β Actin | Mouse | MAB1501 | Merck Millipore | 1:1000 |
| 2',3'-cyclic nucleotide 3' phosphodiesterase (CNPase) | Rabbit | SC-30158 | Santa Cruz | 1:200 |
| Myelin proteolipid protein (PLPC1) | Mouse | MAB388 | Merck Millipore | 1:300 |
| Neuronal nuclear (NeuN) | Mouse | MAB377 | Merck Millipore | 1:1000 |
| Glial fibrillary acidic protein (GFAP) | Rabbit | Z0334 | DAKO | 1:3000 |
| Ionized calcium-binding adapter molecule 1 (Iba1) | Goat | SC-28530 | Santa Cruz | 1:300 |
| Oligodendrocyte transcription factor 2 (Olig2) | Mouse | MABN50 | Merck Millipore | 1:200 |
| Goat IgG-horseradish peroxidase | Rabbit | SC-2030 | Santa Cruz | 1:1000 |
| Goat IgG-horseradish peroxidase | Mouse | SC-2005 | Santa Cruz | 1:2000 |
| Goat IgG (H+L) Alexa Fluor® 488 conjugate | Mouse | A11029 | Thermo Fisher Scientific | 1:500 |
| Goat IgG (H+L) Alexa Fluor® 594 conjugate | Rabbit | A11037 | Thermo Fisher Scientific | 1:500 |
| Donkey IgG (H+L) Alexa Fluor® 594 conjugate | Goat | A11058 | Thermo Fisher Scientific | 1:500 |

Supplementary Table 2. Primer sequences

| **Gene name and symbol** | **Forward primer** | **Reverse primer** |
| --- | --- | --- |
| Actin beta *Actb* | CTGGCACCACACCTTCTA | GGTGGTGAAGCTGTAGCC |
| Glyceraldehyde-3-phosphate dehydrogenase *Gapdh* | CTGGAGAAACCTGCCAAGTA | CGTATTCATTGTCATACCAGG |
| Ribosomal protein L13A *Rpl13a* | GGTGGAAGTACCAGGCAGTGA | TTCCGTAACCTCAAGATCTGCTT |
| Hypoxanthine guanine phosphoribosyl transferase *Hprt* | CGTCGTGATTAGCGATGATGAA | TCCAAATCCTCGGCATAATGAT |
| Phospholipase A2, group VI *Pla2g6* | TCTTGTGCCTCCGGTTTCC | TGGAGGTGAACCCAGGATTG |
| Fatty acid 2-hydroxylase *Fa2h* | ATTACCTCATCATGTTGCATTTTGTC | CCAGACGGGAGCCATCAA |
| ATPase type 13A2 *Atp13a2* | GGCAGGACACCTCCGAACT | TAGCGTTGACCTTGGAGGACATA |
| Chromosome 19 open reading frame 12 *C19orf12* | GCAGGCCCCAGCGTTAA | CCTGGACCTGGGAGATGATAGA |
| Ceruloplasmin *Cp* | CGCTGGCACTGGTTCACA | GACTTTTGATGTTGAGTGCCTTACAA |
| Ferritin heavy chain *Ftl* | AGTCTCTCCAGTCGCAGCCTC | CCCGCGATCGTTCTGAAACTC |

Supplementary Table 3. Aperio ImageScope Positive Pixel Count Algorithm parameters

| **Staining category** | **Aperio density scale** | **Representative color in Mark-up image** |
| --- | --- | --- |
| Negative staining | 0-25 | Dark blue |
| Weak positive staining | 25-75 | Yellow |
| Moderate positive staining | 75-100 | Orange |
| Highly positive staining | 100-255 | Red |

Supplementary Table 4. NBIA related genes investigated in the *Hfe^‑/‑^xTfr2^mut^* mouse brain

| **Gene name and symbol** | **Proposed roles of encoded protein** | **Transcripts fold-change by array**  **(*p* Value)** | **NBIA type** | **Psychiatric abnormality**  **or cognitive impairment** | **Iron abnormality** | **Myelin abnormality** |
| --- | --- | --- | --- | --- | --- | --- |
| Phospholipase A2, group VI *Pla2g6* | Fatty acid release from phospholipids^1^ | ↓1.60 (0.010) | PLAN | Yes^2, 3^ | MRI^4^ | MRI^4^ |
| Fatty acid 2-hydroxylase  *Fa2h* | 2-hydroxy sphingolipid synthesis^5, 6^ | ↓1.41 (0.002) | FAHN | Yes^7^ | MRI^8^ | MRI^8, 9^,  animal study^10, 11^ |
| Ceruloplasmin *Cp* | Oxidation of Fe(II)transferrin to Fe(III) transferrin | ↓1.35 (0.013) | Aceruloplasminemia | Yes^12, 13^ | MRI^14^,  post-mortem^15^,  animal study^16, 17^ | No^17^ |
| Chromosome 19 open reading frame 12 *C19orf12* | Fatty acid biogenesis^18^ | ↓1.28 (0.023) | MPAN | Yes^18-20^ | MRI^18^,  post-mortem^18, 21^ | MRI^18^ |
| ATPase type 13A2 *Atp13a2* | Ceramide synthesis in lysosome^22^, phospholipid distribution in myelin^23, 24^ | ↓1.17 (0.047) | Kufor-Rakeb Disease | Yes^25^ | MRI^26^ | Biopsy^26^ |
| Ferritin, light polypeptide *Ftl* | Iron storage | *↑2.3  (0.0005) | Ferritinopathy | Yes^27-29^ | MRI^30^,  animal model^31, 32^ | Post-mortem^33^ |
| Pantothenate kinase 2 *Pank2* | Biosynthesis of CoA^34^ | NS | PKAN | Yes^35-37^ | MRI^38^,  post-mortem^39^ | No^40^ |
| CoA synthase *Coasy* | Biosynthesis of CoA from pantothenic acid^41^ | NS | CoPAN | Yes^42, 43^ | MRI^42^ | NA |
| WD repeat domain 45 *Wdr45* | Autophagy^44^ | NS | BPAN | Yes^45-47^ | MRI^48^ | MRI^48, 49^ |
| DDB1 and CUL4 associated factor 17 *Dcaf17* | Ubiquitin ligase^50^ | NS | Woodhouse-Sakati syndrome | Yes^51, 52^ | MRI^53^ | MRI^54, 55^ |

Data are presented as fold-change and associated *p* value in brackets, NS: non-significant (*p*>0.05), NA: no reported data available, * by Western immunoblotting.

Supplementary Table 5. Myelin-linked genes differentially expressed in the *Hfe^‑/‑^xTfr2^mut^* mouse brain

| **Gene name and symbol** | **Function of encoded protein** | ***p* Value** | **Transcripts fold-change by array** |
| --- | --- | --- | --- |
| Tumor necrosis factor receptor superfamily, member 21 *Tnfrsf21* | Negatively regulates oligodendrocyte survival, maturation and myelination^56^ | 0.002 | ↓1.50 |
| Myelin-associated oligodendrocytic basic protein *Mobp* | Compacting/ stabilizing the myelin sheath, major myelin component^57, 58^ | 0.0004 | ↓1.48 |
| Sphingosine-1-phosphate phosphotase 2 *Sgpp2* | Catalysing the degradation of Sphingosine-1-phosphate regenerating sphingosine^59^ | 0.0001 | ↓1.40 |
| Neutral sphingomyelinase (N-SMase) activation associated factor *Nsmaf* | Activation of neutral sphingomyelinase^60^ | 0.01 | ↓1.34 |
| Reticulon 4 *Rtn4* | Myelin outgrowth inhibitor^61-63^ | 0.034 | ↓1.33 |
| Transferrin *Tf* | Iron transport and *Mbp* transcription regulator^64, 65^ | 0.00002 | ↓1.33 |
| Gap junction protein, gamma 2 *Gjc2* | Myelination^66, 67^ | 0.005 | ↓1.27 |
| Sphingomyelin phosphodiesterase *Smpd1* | Converting sphingomyelin to ceramide^68^ | 0.0001 | ↓1.25 |
| Histamine receptor H3 *Hrh3* | Myelin repair and remyelination^69-71^ | 0.041 | ↓1.25 |
| Tetraspanin 2 *Tspan2* | Oligodendrocyte differentiation to myelin-forming glia/stabilizing mature myelin^72-74^ | 0.023 | ↓1.22 |
| Ring finger protein 10 *Rnf10* | Transcriptional regulation of the myelin-associated glycoprotein gene^75^ | 0.044 | ↓1.21 |
| Leucine rich repeat and Ig domain containing 1 *Lingo1* | Negatively regulates the myelination^76, 77^ | 0.016 | ↓1.20 |
| 2',3'-cyclic nucleotide 3' phosphodiesterase *Cnp* | Hydrolyzing 2′,3′-cyclic nucleotides to their 2′ derivatives, major myelin component^78^ | 0.009 | ↓1.18 |
| Myelin oligodendrocyte glycoprotein *Mog* | Myelin maintenance^79, 80^ | 0.039 | ↓1.14 |
| Ganglioside induced differentiation-associated protein 1 *Gdap1* | Myelination^81-83^ | 0.038 | ↓1.13 |
| Claudin 11 *Cldn11* | Myelination^84^ | 0.012 | ↓1.13 |

Data are presented as fold-change and associated *p* value.

Supplementary Table 6. Myelin-linked genes differentially expressed in basal ganglia of NBIA cases.

| **Gene name and symbol** | **NBIA case 1**  **Fold-change** | **NBIA case 2**  **Fold-change** |
| --- | --- | --- |
| Tumor necrosis factor receptor superfamily, member 21 *TNFRSF21* | ↓4.25 | ↓1.85 |
| Myelin↓associated oligodendrocytic basic protein *MOBP* | ↓2.6 | ↓2.83 |
| Gap junction protein, gamma 2 *GJC2* | ↓2.47 | ↓2.87 |
| Superoxide dismutase 1, soluble *SOD1* | ↓2.31 | ↑1.52 |
| Reticulon 4 *RTN4* | ↓2.17 | ↑3.09 |
| Myelin transcription factor 1-like *MYT1L* | ↓2.15 | NS |
| Myotubularin related protein 2 *MTMR2* | ↓1.58 | NS |
| Neutral sphingomyelinase (N-SMase) activation associated factor *NSMAF* | ↑1.52 | ↑2.36 |
| Myelin protein zero-like 2 *MPZL2* | ↑1.66 | NS |
| Annexin A2 *ANXA2* | ↑1.7 | ↑1.6 |
| Myelin oligodendrocyte glycoprotein *MOG* | ↑1.8 | ↑2.44 |
| Oligodendrocyte myelin glycoprotein *OMG* | ↑1.89 | ↑2.35 |
| Ring finger protein 10 *RNF10* | ↑1.91 | NS |
| Sphingosine-1-phosphate phosphotase 2 *SGPP2* | ↑1.91 | ↑2.06 |
| Peripheral myelin protein 22 *PMP22* | ↑1.93 | NS |
| Similar to Sphingomyelin phosphodiesterase precursor *LOC654116* | ↑2.01 | NS |
| Myelin basic protein *MBP* | ↑2.33 | ↑1.82 |
| Sphingomyelin phosphodiesterase 1, acid lysosomal *SMPD1* | ↑2.4 | NS |
| Myelin-associated glycoprotein *MAG* | ↑2.41 | ↑2.44 |
| Oligodendrocytic myelin paranodal and inner loop protein *OPALIN* | ↑2.7 | ↑3.3 |
| Peripheral myelin protein 2 *PMP2* | NS | ↑1.61 |
| Myelin transcription factor 1 *MYT1* | NS | ↓3.7 |

Data are presented as fold-change, NS: non-significant (FC ≤1.5).

Supplementary Figure 1. Brain molecular pathways potentially affected by iron loading. Significantly enriched pathways (*p*<0.05) were determined using proprietorial methods by A) DAVID or B) GATHER within the intersection and union sets of differentially-expressed genes in the *Hfe^‑/‑^xTfr2^mut^* mouse brain or C) determined by DAVID from the set of overlapping transcripts between the *Hfe^‑/‑^xTfr2^mut^* mouse and the NBIA brains.

1. Engel LA, Jing Z, O'Brien DE, Sun M, Kotzbauer PT. Catalytic function of PLA2G6 is impaired by mutations associated with infantile neuroaxonal dystrophy but not dystonia-parkinsonism. *PLoS One* 2010; **5**(9)**:** e12897.

2. Gregory A, Westaway SK, Holm IE, Kotzbauer PT, Hogarth P, Sonek S *et al.* Neurodegeneration associated with genetic defects in phospholipase A(2). *Neurology* 2008; **71**(18)**:** 1402-1409.

3. Paisan-Ruiz C, Bhatia KP, Li A, Hernandez D, Davis M, Wood NW *et al.* Characterization of PLA2G6 as a locus for dystonia-parkinsonism. *Ann Neurol* 2009; **65**(1)**:** 19-23.

4. Illingworth MA, Meyer E, Chong WK, Manzur AY, Carr LJ, Younis R *et al.* PLA2G6-associated neurodegeneration (PLAN): further expansion of the clinical, radiological and mutation spectrum associated with infantile and atypical childhood-onset disease. *Mol Genet Metab* 2014; **112**(2)**:** 183-189.

5. Hama H. Fatty acid 2-Hydroxylation in mammalian sphingolipid biology. *Biochim Biophys Acta* 2010; **1801**(4)**:** 405-414.

6. Eckhardt M, Yaghootfam A, Fewou SN, Zoller I, Gieselmann V. A mammalian fatty acid hydroxylase responsible for the formation of alpha-hydroxylated galactosylceramide in myelin. *Biochem J* 2005; **388**(Pt 1)**:** 245-254.

7. Garone C, Pippucci T, Cordelli DM, Zuntini R, Castegnaro G, Marconi C *et al.* FA2H-related disorders: a novel c.270+3A>T splice-site mutation leads to a complex neurodegenerative phenotype. *Dev Med Child Neurol* 2011; **53**(10)**:** 958-961.

8. Kruer MC, Boddaert N, Schneider SA, Houlden H, Bhatia KP, Gregory A *et al.* Neuroimaging features of neurodegeneration with brain iron accumulation. *AJNR Am J Neuroradiol* 2012; **33**(3)**:** 407-414.

9. Kruer MC, Paisan-Ruiz C, Boddaert N, Yoon MY, Hama H, Gregory A *et al.* Defective FA2H leads to a novel form of neurodegeneration with brain iron accumulation (NBIA). *Ann Neurol* 2010; **68**(5)**:** 611-618.

10. Potter KA, Kern MJ, Fullbright G, Bielawski J, Scherer SS, Yum SW *et al.* Central nervous system dysfunction in a mouse model of FA2H deficiency. *Glia* 2011; **59**(7)**:** 1009-1021.

11. Zoller I, Meixner M, Hartmann D, Bussow H, Meyer R, Gieselmann V *et al.* Absence of 2-hydroxylated sphingolipids is compatible with normal neural development but causes late-onset axon and myelin sheath degeneration. *J Neurosci* 2008; **28**(39)**:** 9741-9754.

12. Skidmore FM, Drago V, Foster P, Schmalfuss IM, Heilman KM, Streiff RR. Aceruloplasminaemia with progressive atrophy without brain iron overload: treatment with oral chelation. *J Neurol Neurosurg Psychiatry* 2008; **79**(4)**:** 467-470.

13. Miyajima H. Aceruloplasminemia. In: Pagon RA, Adam MP, Ardinger HH, Wallace SE, Amemiya A, Bean LJH *et al.* (eds). *GeneReviews(R)*. University of Washington, Seattle: Seattle WA, 1993.

14. Loreal O, Turlin B, Pigeon C, Moisan A, Ropert M, Morice P *et al.* Aceruloplasminemia: new clinical, pathophysiological and therapeutic insights. *J Hepatol* 2002; **36**(6)**:** 851-856.

15. Kaneko K, Yoshida K, Arima K, Ohara S, Miyajima H, Kato T *et al.* Astrocytic deformity and globular structures are characteristic of the brains of patients with aceruloplasminemia. *J Neuropathol Exp Neurol* 2002; **61**(12)**:** 1069-1077.

16. Jiang R, Hua C, Wan Y, Jiang B, Hu H, Zheng J *et al.* Hephaestin and Ceruloplasmin Play Distinct but Interrelated Roles in Iron Homeostasis in Mouse Brain. *J Nutr* 2015.

17. Jeong SY, David S. Age-related changes in iron homeostasis and cell death in the cerebellum of ceruloplasmin-deficient mice. *J Neurosci* 2006; **26**(38)**:** 9810-9819.

18. Hartig MB, Iuso A, Haack T, Kmiec T, Jurkiewicz E, Heim K *et al.* Absence of an orphan mitochondrial protein, c19orf12, causes a distinct clinical subtype of neurodegeneration with brain iron accumulation. *Am J Hum Genet* 2011; **89**(4)**:** 543-550.

19. Dogu O, Krebs CE, Kaleagasi H, Demirtas Z, Oksuz N, Walker RH *et al.* Rapid disease progression in adult-onset mitochondrial membrane protein-associated neurodegeneration. *Clin Genet* 2013; **84**(4)**:** 350-355.

20. Deschauer M, Gaul C, Behrmann C, Prokisch H, Zierz S, Haack TB. C19orf12 mutations in neurodegeneration with brain iron accumulation mimicking juvenile amyotrophic lateral sclerosis. *J Neurol* 2012; **259**(11)**:** 2434-2439.

21. Horvath R. Brain iron takes off: a new propeller protein links neurodegeneration with autophagy. *Brain* 2013; **136**(Pt 6)**:** 1687-1691.

22. Nixon RA, Yang DS, Lee JH. Neurodegenerative lysosomal disorders: a continuum from development to late age. *Autophagy* 2008; **4**(5)**:** 590-599.

23. van Veen S, Sorensen DM, Holemans T, Holen HW, Palmgren MG, Vangheluwe P. Cellular function and pathological role of ATP13A2 and related P-type transport ATPases in Parkinson's disease and other neurological disorders. *Front Mol Neurosci* 2014; **7:** 48.

24. Sebastian TT, Baldridge RD, Xu P, Graham TR. Phospholipid flippases: building asymmetric membranes and transport vesicles. *Biochim Biophys Acta* 2012; **1821**(8)**:** 1068-1077.

25. Crosiers D, Ceulemans B, Meeus B, Nuytemans K, Pals P, Van Broeckhoven C *et al.* Juvenile dystonia-parkinsonism and dementia caused by a novel ATP13A2 frameshift mutation. *Parkinsonism Relat Disord* 2011; **17**(2)**:** 135-138.

26. Paisan-Ruiz C, Guevara R, Federoff M, Hanagasi H, Sina F, Elahi E *et al.* Early-onset L-dopa-responsive parkinsonism with pyramidal signs due to ATP13A2, PLA2G6, FBXO7 and spatacsin mutations. *Mov Disord* 2010; **25**(12)**:** 1791-1800.

27. Maciel P, Cruz VT, Constante M, Iniesta I, Costa MC, Gallati S *et al.* Neuroferritinopathy: missense mutation in FTL causing early-onset bilateral pallidal involvement. *Neurology* 2005; **65**(4)**:** 603-605.

28. Wills AJ, Sawle GV, Guilbert PR, Curtis AR. Palatal tremor and cognitive decline in neuroferritinopathy. *J Neurol Neurosurg Psychiatry* 2002; **73**(1)**:** 91-92.

29. Curtis AR, Fey C, Morris CM, Bindoff LA, Ince PG, Chinnery PF *et al.* Mutation in the gene encoding ferritin light polypeptide causes dominant adult-onset basal ganglia disease. *Nat Genet* 2001; **28**(4)**:** 350-354.

30. Chinnery PF, Crompton DE, Birchall D, Jackson MJ, Coulthard A, Lombes A *et al.* Clinical features and natural history of neuroferritinopathy caused by the FTL1 460InsA mutation. *Brain* 2007; **130**(Pt 1)**:** 110-119.

31. Vidal R, Miravalle L, Gao X, Barbeito AG, Baraibar MA, Hekmatyar SK *et al.* Expression of a mutant form of the ferritin light chain gene induces neurodegeneration and iron overload in transgenic mice. *J Neurosci* 2008; **28**(1)**:** 60-67.

32. Maccarinelli F, Pagani A, Cozzi A, Codazzi F, Di Giacomo G, Capoccia S *et al.* A novel neuroferritinopathy mouse model (FTL 498InsTC) shows progressive brain iron dysregulation, morphological signs of early neurodegeneration and motor coordination deficits. *Neurobiol Dis* 2014.

33. Mancuso M, Davidzon G, Kurlan RM, Tawil R, Bonilla E, Di Mauro S *et al.* Hereditary ferritinopathy: a novel mutation, its cellular pathology, and pathogenetic insights. *J Neuropathol Exp Neurol* 2005; **64**(4)**:** 280-294.

34. Abiko Y. Investigations on pantothenic acid and its related compounds. IX. Biochemical studies.4. Separation and substrate specificity of pantothenate kinase and phosphopantothenoylcysteine synthetase. *J Biochem* 1967; **61**(3)**:** 290-299.

35. del Valle-Lopez P, Perez-Garcia R, Sanguino-Andres R, Gonzalez-Pablos E. Adult onset Hallervorden-Spatz disease with psychotic symptoms. *Actas Esp Psiquiatr* 2011; **39**(4)**:** 260-262.

36. Pellecchia MT, Valente EM, Cif L, Salvi S, Albanese A, Scarano V *et al.* The diverse phenotype and genotype of pantothenate kinase-associated neurodegeneration. *Neurology* 2005; **64**(10)**:** 1810-1812.

37. Kumar N, Boes CJ, Babovic-Vuksanovic D, Boeve BF. The "eye-of-the-tiger" sign is not pathognomonic of the PANK2 mutation. *Arch Neurol* 2006; **63**(2)**:** 292-293.

38. Morales-Briceno H, Chacon-Camacho OF, Perez-Gonzalez EA, Arteaga-Vazquez J, Rodriguez-Violante M, Cervantes-Arriaga A *et al.* Clinical, imaging, and molecular findings in a sample of Mexican families with pantothenate kinase-associated neurodegeneration. *Clin Genet* 2015; **87**(3)**:** 259-265.

39. Kruer MC, Hiken M, Gregory A, Malandrini A, Clark D, Hogarth P *et al.* Novel histopathologic findings in molecularly-confirmed pantothenate kinase-associated neurodegeneration. *Brain* 2011; **134**(Pt 4)**:** 947-958.

40. Brunetti D, Dusi S, Morbin M, Uggetti A, Moda F, D'Amato I *et al.* Pantothenate kinase-associated neurodegeneration: altered mitochondria membrane potential and defective respiration in Pank2 knock-out mouse model. *Hum Mol Genet* 2012; **21**(24)**:** 5294-5305.

41. Daugherty M, Polanuyer B, Farrell M, Scholle M, Lykidis A, de Crecy-Lagard V *et al.* Complete reconstitution of the human coenzyme A biosynthetic pathway via comparative genomics. *J Biol Chem* 2002; **277**(24)**:** 21431-21439.

42. Dusi S, Valletta L, Haack T  B, Tsuchiya Y, Venco P, Pasqualato S *et al.* Exome Sequence Reveals Mutations in CoA Synthase as a Cause of Neurodegeneration with Brain Iron Accumulation. *Am J Hum Genet* 2014; **94**(1)**:** 11-22.

43. Dusi S, Valletta L, Haack TB, Tsuchiya Y, Venco P, Pasqualato S *et al.* Exome sequence reveals mutations in CoA synthase as a cause of neurodegeneration with brain iron accumulation. *Am J Hum Genet* 2014; **94**(1)**:** 11-22.

44. Tsuyuki S, Takabayashi M, Kawazu M, Kudo K, Watanabe A, Nagata Y *et al.* Detection of WIPI1 mRNA as an indicator of autophagosome formation. *Autophagy* 2014; **10**(3)**:** 497-513.

45. Verhoeven WM, Egger JI, Koolen DA, Yntema H, Olgiati S, Breedveld GJ *et al.* Beta-propeller protein-associated neurodegeneration (BPAN), a rare form of NBIA: novel mutations and neuropsychiatric phenotype in three adult patients. *Parkinsonism Relat Disord* 2014; **20**(3)**:** 332-336.

46. Nishioka K, Oyama G, Yoshino H, Li Y, Matsushima T, Takeuchi C *et al.* High frequency of beta-propeller protein-associated neurodegeneration (BPAN) among patients with intellectual disability and young-onset parkinsonism. *Neurobiol Aging* 2015; **36**(5)**:** 2004 e2009-2004 e2015.

47. Saitsu H, Nishimura T, Muramatsu K, Kodera H, Kumada S, Sugai K *et al.* De novo mutations in the autophagy gene WDR45 cause static encephalopathy of childhood with neurodegeneration in adulthood. *Nat Genet* 2013; **45**(4)**:** 445-449, 449e441.

48. Rathore GS, Schaaf CP, Stocco AJ. Novel mutation of the WDR45 gene causing beta-propeller protein-associated neurodegeneration. *Mov Disord* 2014; **29**(4)**:** 574-575.

49. Okamoto N, Ikeda T, Hasegawa T, Yamamoto Y, Kawato K, Komoto T *et al.* Early manifestations of BPAN in a pediatric patient. *Am J Med Genet A* 2014; **164**(12)**:** 3095-3099.

50. Lee J, Zhou P. DCAFs, the missing link of the CUL4-DDB1 ubiquitin ligase. *Mol Cell* 2007; **26**(6)**:** 775-780.

51. Steindl K, Alazami AM, Bhatia KP, Wuerfel JT, Petersen D, Cartolari R *et al.* A novel C2orf37 mutation causes the first Italian cases of Woodhouse Sakati syndrome. *Clin Genet* 2010; **78**(6)**:** 594-597.

52. Devriendt K, Legius E, Fryns JP. Progressive extrapyramidal disorder with primary hypogonadism and alopecia in sibs: a new syndrome? *Am J Med Genet* 1996; **62**(1)**:** 54-57.

53. Levi S, Finazzi D. Neurodegeneration with brain iron accumulation: update on pathogenic mechanisms. *Front Pharmacol* 2014; **5:** 99.

54. Alazami AM, Schneider SA, Bonneau D, Pasquier L, Carecchio M, Kojovic M *et al.* C2orf37 mutational spectrum in Woodhouse-Sakati syndrome patients. *Clin Genet* 2010; **78**(6)**:** 585-590.

55. Ben-Omran T, Ali R, Almureikhi M, Alameer S, Al-Saffar M, Walsh CA *et al.* Phenotypic heterogeneity in Woodhouse-Sakati syndrome: two new families with a mutation in the C2orf37 gene. *Am J Med Genet A* 2011; **155A**(11)**:** 2647-2653.

56. Mi S, Lee X, Hu Y, Ji B, Shao Z, Yang W *et al.* Death receptor 6 negatively regulates oligodendrocyte survival, maturation and myelination. *Nat Med* 2011; **17**(7)**:** 816-821.

57. Yamamoto Y, Yoshikawa H, Nagano S, Kondoh G, Sadahiro S, Gotow T *et al.* Myelin-associated oligodendrocytic basic protein is essential for normal arrangement of the radial component in central nervous system myelin. *Eur J Neurosci* 1999; **11**(3)**:** 847-855.

58. Han H, Myllykoski M, Ruskamo S, Wang C, Kursula P. Myelin-specific proteins: A structurally diverse group of membrane-interacting molecules. *BioFactors* 2013; **39**(3)**:** 233-241.

59. Ogawa C, Kihara A, Gokoh M, Igarashi Y. Identification and characterization of a novel human sphingosine-1-phosphate phosphohydrolase, hSPP2. *J Biol Chem* 2003; **278**(2)**:** 1268-1272.

60. Tcherkasowa AE, Adam-Klages S, Kruse ML, Wiegmann K, Mathieu S, Kolanus W *et al.* Interaction with factor associated with neutral sphingomyelinase activation, a WD motif-containing protein, identifies receptor for activated C-kinase 1 as a novel component of the signaling pathways of the p55 TNF receptor. *J Immunol* 2002; **169**(9)**:** 5161-5170.

61. Domeniconi M, Cao Z, Spencer T, Sivasankaran R, Wang KC, Nikulina E *et al.* Myelin-Associated Glycoprotein Interacts with the Nogo66 Receptor to Inhibit Neurite Outgrowth. *Neuron* 2002; **35**(2)**:** 283-290.

62. GrandPre T, Nakamura F, Vartanian T, Strittmatter SM. Identification of the Nogo inhibitor of axon regeneration as a Reticulon protein. *Nature* 2000; **403**(6768)**:** 439-444.

63. Chen MS, Huber AB, van der Haar ME, Frank M, Schnell L, Spillmann AA *et al.* Nogo-A is a myelin-associated neurite outgrowth inhibitor and an antigen for monoclonal antibody IN-1. *Nature* 2000; **403**(6768)**:** 434-439.

64. Espinosa-Jeffrey A, Kumar S, Zhao PM, Awosika O, Agbo C, Huang A *et al.* Transferrin regulates transcription of the MBP gene and its action synergizes with IGF-1 to enhance myelinogenesis in the md rat. *Dev Neurosci* 2002; **24**(2-3)**:** 227-241.

65. Espinosa de los Monteros A, Kumar S, Zhao P, Huang CJ, Nazarian R, Pan T *et al.* Transferrin is an essential factor for myelination. *Neurochem Res* 1999; **24**(2)**:** 235-248.

66. Odermatt B, Wellershaus K, Wallraff A, Seifert G, Degen J, Euwens C *et al.* Connexin 47 (Cx47)-deficient mice with enhanced green fluorescent protein reporter gene reveal predominant oligodendrocytic expression of Cx47 and display vacuolized myelin in the CNS. *J Neurosci* 2003; **23**(11)**:** 4549-4559.

67. Sargiannidou I, Markoullis K, Kleopa KA. Molecular mechanisms of gap junction mutations in myelinating cells. *Histol Histopathol* 2010; **25**(9)**:** 1191-1206.

68. Stoffel W. Functional analysis of acid and neutral sphingomyelinases in vitro and in vivo. *Chem Phys Lipids* 1999; **102**(1-2)**:** 107-121.

69. Saligrama N, Noubade R, Case LK, del Rio R, Teuscher C. Combinatorial roles for histamine H(1)–H(2) and H(3)–H(4) receptors in autoimmune inflammatory disease of the central nervous system. *Eur J Immunol* 2012; **42**(6)**:** 1536-1546.

70. Gillson G, Wright JV, DeLack E, Ballasiotes G. Transdermal histamine in multiple sclerosis: part one -- clinical experience. *Altern Med Rev* 1999; **4**(6)**:** 424-428.

71. Gillson G, Wright JV, DeLack E, Ballasiotes G. Transdermal histamine in multiple sclerosis, part two: a proposed theoretical basis for its use. *Altern Med Rev* 2000; **5**(3)**:** 224-248.

72. de Monasterio-Schrader P, Patzig J, Möbius W, Barrette B, Wagner TL, Kusch K *et al.* Uncoupling of neuroinflammation from axonal degeneration in mice lacking the myelin protein tetraspanin-2. *Glia* 2013; **61**(11)**:** 1832-1847.

73. Birling MC, Tait S, Hardy RJ, Brophy PJ. A novel rat tetraspan protein in cells of the oligodendrocyte lineage. *J Neurochem* 1999; **73**(6)**:** 2600-2608.

74. Teuscher C, Subramanian M, Noubade R, Gao JF, Offner H, Zachary JF *et al.* Central histamine H3 receptor signaling negatively regulates susceptibility to autoimmune inflammatory disease of the CNS. *Proc Natl Acad Sci U S A* 2007; **104**(24)**:** 10146-10151.

75. Hoshikawa S, Ogata T, Fujiwara S, Nakamura K, Tanaka S. A novel function of RING finger protein 10 in transcriptional regulation of the myelin-associated glycoprotein gene and myelin formation in Schwann cells. *PLoS One* 2008; **3**(10)**:** e3464.

76. Mi S, Miller RH, Lee X, Scott ML, Shulag-Morskaya S, Shao Z *et al.* LINGO-1 negatively regulates myelination by oligodendrocytes. *Nat Neurosci* 2005; **8**(6)**:** 745-751.

77. Rudick RA, Mi S, Sandrock AW, Jr. LINGO-1 antagonists as therapy for multiple sclerosis: in vitro and in vivo evidence. *Expert Opin Biol Ther* 2008; **8**(10)**:** 1561-1570.

78. Morell P, Quarles, R.H., Norton, W. Formation, structure and biochemistry of myelin. In: G. Siegel BA, R.W. Albers, P. Molinoff (ed). *Basic Neurochemistry*. Raven Press: New York, 1989.

79. Johns TG, Bernard CC. The structure and function of myelin oligodendrocyte glycoprotein. *J Neurochem* 1999; **72**(1)**:** 1-9.

80. Roth MP, Malfroy L, Offer C, Sevin J, Enault G, Borot N *et al.* The human myelin oligodendrocyte glycoprotein (MOG) gene: complete nucleotide sequence and structural characterization. *Genomics* 1995; **28**(2)**:** 241-250.

81. Shield AJ, Murray TP, Board PG. Functional characterisation of ganglioside-induced differentiation-associated protein 1 as a glutathione transferase. *Biochem Biophys Res Commun* 2006; **347**(4)**:** 859-866.

82. Nelis E, Erdem S, Van Den Bergh PY, Belpaire-Dethiou MC, Ceuterick C, Van Gerwen V *et al.* Mutations in GDAP1: autosomal recessive CMT with demyelination and axonopathy. *Neurology* 2002; **59**(12)**:** 1865-1872.

83. Baxter RV, Ben Othmane K, Rochelle JM, Stajich JE, Hulette C, Dew-Knight S *et al.* Ganglioside-induced differentiation-associated protein-1 is mutant in Charcot-Marie-Tooth disease type 4A/8q21. *Nat Genet* 2002; **30**(1)**:** 21-22.

84. Chow E, Mottahedeh J, Prins M, Ridder W, Nusinowitz S, Bronstein JM. Disrupted compaction of CNS myelin in an OSP/Claudin-11 and PLP/DM20 double knockout mouse. *Mol Cell Neurosci* 2005; **29**(3)**:** 405-413.
